# National survey on clinical and genetic characteristics of patients with hereditary angioedema in Latvia

**DOI:** 10.1186/s13223-023-00783-6

**Published:** 2023-04-08

**Authors:** Adine Kanepa, Inga Nartisa, Dmitrijs Rots, Linda Gailite, Henriette Farkas, Natalja Kurjane

**Affiliations:** 1 Riga Stradiņš University, Dzirciema Street 16, Riga, LV-1007 Latvia; 2 Children’s Clinical University Hospital, Riga, Latvia; 3 Department of Internal Medicine and Haematology, Hungarian Angioedema Center of Reference and Excellence, Semmelweis University, Budapest, Hungary; 4 Pauls Stradiņš Clinical University Hospital, Riga, Latvia

**Keywords:** Hereditary angioedema, C1-inhibitor deficiency, *SERPING1* gene

## Abstract

**Background:**

Hereditary angioedema (HAE) is a rare and life-threatening inborn error of immunity. HAE is mostly caused by pathogenic variations in the serine protease inhibitor gene 1 (*SERPING1*), leading to deficient or dysfunctional C1-inhibitor (C1-INH), overproduction of bradykinin, and development of recurrent subcutaneous and/or submucosal oedema. The prevalence of HAE is 1 in 50,000 − 100000 people worldwide. We aimed to describe the clinical features and genetic spectrum of hereditary angioedema with C1-INH deficiency (C1-INH-HAE) in Latvia.

**Methods:**

All patients from Latvia diagnosed with HAE (types I/II) from 2006 to March 2022 were included in the study. Laboratory tests and clinical data were analysed, and genetic tests with Sanger sequencing and whole genome sequencing were performed.

**Results:**

The study identified 10 C1-INH-HAE patients (nine females, one male) from eight families. The point prevalence of HAE in Latvia is 0.53 per 100 000 inhabitants. Of all patients, seven (70%) had HAE type I and three (30%) had HAE type II. The median age of patients was 54 years and the median age at onset of symptoms was 15 years. A significant delay (median 20.5 years) until diagnosis was observed, and 60% of patients had a positive family history of angioedema. All HAE patients have been hospitalised a median two times during their lifetime. Skin (100%), abdominal (80%), and airway (80%) oedema were the most frequent symptoms. Triggering factors (60%) and prodromal symptoms (90%) were referred. Attacks were severe in 50% of patients, moderate in 10%, and mild in 40%. Pathogenic variations of *SERPING1* were identified in eight patients (six families), confirming the diagnosis molecularly. In two patients (two families), no pathogenic variations in the genes were found even after whole genome sequencing.

**Conclusions:**

Current data shows a significant delay and clear underdiagnosis of HAE in Latvia. Higher awareness and better information and communication between doctors would improve the diagnosis and management of HAE; as would screening of family members, patients with recurrent angioedema unresponsive to antihistamines and glucocorticoids, and patients with recurrent episodes of severe, unexplained abdominal pain.

**Supplementary Information:**

The online version contains supplementary material available at 10.1186/s13223-023-00783-6.

## Background

Hereditary angioedema (HAE) is a rare, life-threatening, autosomal dominant inborn error of immunity [1, 2]. There are 3 types of HAE: type I (85%), II (15%), and n-C1-INH HAE, which can be distinguished by levels and functional activity of a C1-INH (Table 1) [2–4]. The prevalence of HAE is 1 in 50,000 − 100,000 people worldwide [1].

**Table 1:** Typical diagnostic laboratory profile of HAE patients (5)

	C1-INH concentration	C1-INH function	C4 protein level
Type I HAE	Low	Low	Low
Type II HAE	Normal/high	Low	Low
n-C1-INH HAE	Normal	Normal	Normal

Clinically, HAE is characterised by recurrent episodes of nonpruritic, nonpitting subcutaneous and/or submucosal oedema lasting for 2 − 5 days, involving mostly the extremities, face, upper airway, and gastrointestinal tract [2, 3, 6, 7]. Laryngeal oedema occurs in 50% of patients and is the most serious complication, which can become life threatening [7]. Patients may have complaints of severe abdominal pain, mimicking acute abdomen and leading to unnecessary surgery [6, 8]. Patients may also report a transient tingling feeling of pressure and pain in the extremities or urogenital pain caused by vascular congestion [6]. Fluid shifts into the interstitial space or peritoneal cavity can cause hypotension and tachycardia [6].

Symptoms usually begin in childhood, worsen at puberty, and become more severe during adolescence. The frequency of attacks varies from weekly episodes to one episode over several years [3]. Attacks of HAE may be spontaneous or triggered by trauma, surgical or dental procedures, emotional stress, menstruation, infection, oestrogen replacement therapies or angiotensin converting enzyme inhibitor (ACEI) [9]. Up to 50% of HAE attacks may be preceded by a prodrome, including fatigue, tightness, tingling, dizziness, malaise, irritability, anxiety, muscle aches and weakness, nausea, and typical rash of HAE (*erythema marginatum)* [3, 6, 10].

Although most HAE patients have a positive family history of angioedema, approximately 25% are due to spontaneous mutations [2, 7, 11].

C1-INH-HAE (types I and II) are mainly caused by pathogenic variations of *SERPING1* that results in deficient or dysfunctional C1-INH, kinin, and/or contact system dysregulation, leading to overproduction of bradykinin, localised vasodilatation, vascular leakage, and the development of massive local oedema [1, 2, 11].

The diagnosis of HAE should be considered in patients with recurrent swelling episodes not responsive to antihistamines or glucocorticoids, unclear abdominal pain episodes, and positive family history of HAE [3].

Most patients with C1-INH-HAE have low complement C4 levels, low C1-INH functional activity, and low (HAE type I) or normal C1-INH level (HAE type II) [12].

HAE management includes treatment of HAE attacks, long-term prophylaxis, and short-term prophylaxis [3, 13, 14]. Long-term prophylaxis should be considered in symptomatic patients whose condition is not controlled with optimal on-demand treatment [3, 13]. Short-term prophylaxis is used to prevent angioedema episodes before and after predictable risk factors [3, 13].

This was the first ever nationwide survey on HAE in Latvia. We aimed to describe the clinical features and genetic spectrum of C1-INH-HAE in Latvia, as data on HAE in the Baltic States have so far not been available.

## Materials and methods

### Patients

As of June 2020, we had sparse data on the prevalence of HAE in Latvia. A survey was initiated among local HAE experts (allergists and immunologists) and among physicians from other disciplines who were known to be aware of HAE (dermatologists, paediatricians, general practitioners, and others). Doctors were contacted via email or phone call and requested to participate in a short survey concerning HAE patients identified by them. To inform the population of Latvia about HAE and its clinical diagnostics and treatment, the national HAE website was created and information about the disease was distributed in Internet media [15]. Patients diagnosed with C1-INH-HAE in Latvia from 2006 to March 2022 were included in the study. Diagnosis of HAE was based on personal and family history of angioedema, and on complement C4 levels, C1-INH levels, C1-INH functional activity, and genetic findings according to the global WAO/EAACI guideline definition 2021 [5].

### Latvian population

Data on demographic characteristics of the Latvian general population were collected from the Central Statistical Bureau of Latvia (March 2022) [16]. The point prevalence was expressed as alive patients per 100,000 inhabitants at a particular point in time (March 2022).

### Data collection

We asked for the following data for each patient: gender, initials, date of birth, date of diagnosis. C4 level, C1-INH level, and C1-INH functional activity we determined from peripheral blood serum. Existing patient data were collected from medical records regarding the onset of symptoms, age at diagnosis, and diagnostic delay. Independent families were identified, and the first diagnosed member of a family was considered the index patient. Only patients who were alive at the time of data analysis were included. The term “patients with a positive family history” was defined as more than one affected person in the family. The term “patient without a family history” was defined as the patient being the sole individual in the family with HAE. Only a bradykinin B2 receptor-antagonist (icatibant) and fresh frozen plasma are currently available in Latvia for the treatment of HAE attacks. The term "hospitalization" was defined as HAE-related emergency department visits or hospitalizations with the corresponding diagnosis.

### Ethics

The study was approved by the Latvian Central Medical Ethics Committee (No. 01–29.1/2878, approved on 03/06/2020) and conforms to the principles of the Declaration of Helsinki. All data were anonymised before statistical analysis. All patients or their legal representatives have signed informed consent.

### Laboratory methods

C4 and C1-INH levels were quantified using nephelometry by Atellica NEPH 630 System (Siemens) with N Antiserum to Human C1-Inhibitor as the reagent. C1-INH functional activity was measured using a chromogenic assay by Sysmex CS 2500 (Siemens) with Berichrom C1-Inhibitor as the reagent. Results were normalised as percentage of normal value (C4 normal range 0.12 − 0.36 g/L; functional C1-INH normal range 70 − 130%; C1-INH level normal range 0.21 − 0.39 g/L). C4 level, C1-INH level, and functional activity were performed in Pauls Stradins Clinical University Hospital Joint Laboratory.

### Diagnosis of C1-INH-HAE

Patients were diagnosed as C1-INH-HAE type I, when C4, C1-INH level and functional activity were below normal range, and as type II when C1-INH level was normal, but C4 and C1-INH functional activity were below normal range.

### Genetic testing

Different range genetic testing was performed to test genetic variations of *SERPING1*. Firstly, Sanger sequencing of the gene coding part (using primers from published manuscript with Big Dye Terminator kit 3.0 following manufacturer protocol) was conducted [17–19]. For two cases, exome sequencing (performed using Twist Bioscience reagents at CeGaT medical laboratory) analysis of the single nucleotide variation and copy number variation (CNV) in *SERPING1* were done. In negative two cases, whole genome sequencing was performed at CeGaT medical laboratory, followed by structural intronic variation in *SERPING1*. Pathogenicity of identified variants was checked in the ClinVar database, or if not reported, pathogenicity was observed following American College of Medical Genetics and Genomics (ACMG) criteria [20].

### Statistical analysis

All data were analysed using Microsoft Office Excel and IBM SPSS Statistics for Windows Version 23. Data related to demographic and clinical indicators were analysed using descriptive statistics and parametric/nonparametric analysis, as appropriate. The point prevalence was expressed as live patients per 100000 inhabitants at a particular point in time (March 2022).

## Results

The study identified 10 C1-INH-HAE patients (nine females, one male) from 2006 to March 2022, living in Latvia. According to the epidemiological data, in March 2022 the population was 1870400 inhabitants. The point prevalence of HAE in Latvia yielded 0.53 per 100000 inhabitants. The median age of patients was 54.0 years (IQR: 35–62, range 27 − 66, n = 10) (Table 2).

**Table 2:** Demographic and clinical features of C1-INH-HAE patients

Demographic features and clinical patterns (n = 10)	Findings/years
Mean age, years	54
Gender	
Female	9
Male	1
HAE type	
Type I	7
Type II	3
Family history	
Yes	6
No	4
Median age at onset of symptoms, years	15.0 (range 6–53 years)
Median age at diagnosis, years	45.5 (range 19–56 years)
Median time from symptoms to diagnosis, years	20.5 (range 3–37 years)

### Type of HAE, family history

Of the 10 C1-INH-HAE patients, seven (70%) had HAE type I and three (30%) had HAE type II.

C1q level (normal) and C1-INH autoantibodies (negative) were determined in two HAE type II patients without pathogenic variations in *SERPING*1.

A total of 10 patients were identified in eight unrelated families. Six patients (60%) had positive family history (Table 2). Timeline presentation of clinical characteristics and laboratory test results for each patient with HAE can be seen in Additional file 1: Figs. S1, S2, S3, S4, S5, S6, S7, S8, S9, S10.

### Age at onset and at diagnosis

The median age at onset of symptoms was 15.0 years (IQR: 7–23, range 6 − 53 years, n = 10); for females it was 16.0 years (IQR: 8–25, range 6 − 53 years, n = 9), and for the male it was 6.0 years (n = 1). The median age at diagnosis was 45.5 years (IQR: 26–52, range 19 − 56 years, n = 10); for females it was 49.0 years (IQR: 25–52, range 19 − 56 years, n = 9), and for the male it was 30.0 years (n = 1). The median time duration to diagnosis was 20.5 years (IQR: 11–32, range 3 − 37 years, n = 10); for females it was 17.0 years (IQR: 10–35, range 3 − 37 years, n = 9), and for the male it was 24.0 years (n = 1). No patients were biochemically diagnosed before onset of symptoms (Table 2).

### Clinical manifestation

A summary of clinical manifestation is reported (Table 3).

**Table 3:** Clinical features of HAE patients

Clinical features	Findings
Attacks per year since the diagnosis of HAE (n = 10)	
1–5	4
6–11	2
12–24	0
> 24	4
Frequency of attacks in the last 12 months^a^ (n = 9)	
Mild	4
Moderate	1
Severe	4
Swelling location (n = 10)	
Skin	10
Lips	9
Tongue	8
Abdomen	8
Larynx	8
Urogenital area	3
Prodrome (n = 9)	
Tiredness	8
Paraesthesia/pain	8
Abdominal pain	8
Nausea	3
*Erythema marginatum*	2
Trigger factors (n = 6)	
Stress	4
Trauma	2
Surgical/dental manipulations	2
Infection	1
Menstruation	1

^a^Severe—if the patient had experienced 12 or more attacks during the last 12 months, moderate—4 − 11 attacks, mild—1 − 3 attacks, and asymptomatic—no attacks

During the previous year (2021), one patient had no attacks and nine had attacks of angioedema. Of those who had attacks, we received data on attack frequency. From nine patients, a median of 10 attacks were reported (IQR: 2–48, range 1 − 60, n = 9); females had median 29 attacks (IQR: 3– 48, range 1 − 60, n = 8), and the male had one attack in the previous year. Females had a median 96 total sick days (IQR: 6 –144, range 0 − 240, n = 9), and the male had 5 total sick days per year.

All 10 HAE patients have been hospitalised a median 2.0 times (IQR: 2–8, range 1 − 20, n = 10) during their lifetime.

Concerning redness in areas with skin angioedema, two patients had *erythema marginatum*.

### Treatment

Patients were asked about treatment of HAE attacks. They also graded the treatment effect as none, poor, moderate, or very good (Table 4).

**Table 4:** Number of patients reporting certain acute treatments for HAE attacks

Treatment for HAE attacks	Patients	Effect
Bradykinin B2 receptor-antagonist (icatibant)	9	3
FFP	3	1
Opioids	2	1
NSAIDs	3	1
Glucocorticoids	8	0
Antihistamines	10	0

The median overall treatment effect was rated on a 4-point scale (no = 0, poor = 1, moderate = 2, and very good = 3)

*FFP* fresh frozen plasma, *NSAIDs* non-steroidal anti-inflammatory drugs

Patients were also asked about prophylactic treatment. They graded the treatment effect as none, poor, moderate, or very good. Fresh frozen plasma, antifibrinolytic agent (tranexamic acid), and attenuated androgens (danazol) are currently available in Latvia for long-term or short-term prophylaxis. (Table 5).

**Table 5:** Number of patients reporting certain treatments for prophylactic HAE treatment

Prophylactic treatment	Patients	Effect
FFP	3	1
Antifibrinolytic agent (tranexamic acid)	4	2
Attenuated androgen (danazol)	4	2

The median overall treatment effect was rated on a 4-step scale (no = 0, poor = 1, moderate = 2 and very good = 3)

*FFP* fresh frozen plasma

Timeline presentation of therapy for each patient with HAE can be seen in Additional file 1: Figs. S1, S2, S3, S4, S5, S6, S7, S8, S9, S10.

### Genetic testing

Genetic testing was performed on 10 C1-INH-HAE patients in eight unrelated families. Pathogenic variations of *SERPING1* were identified in eight (80%) patients (six families) with C1-INH-HAE, confirming the diagnosis molecularly. Identified variants shown (Table 6). In two (20%) patients (two families) with C1-INH-HAE, no pathogenic variations in the genes were found even after whole genome sequencing. Family trees for each patient with HAE seen in Additional file 1: Figs. S1, S2, S3, S4, S5, S6, S7, S8, S9, S10.

**Table 6:** Characteristics of the identified *SERPING1* variants of HAE type I and II patients

Patient	Confirmed HAE type	Identified variant in *SERPING1* (reference gene NM_000062.2), dbSNP	Found in n index individuals (n total)	Pathogenicity	Reported before^a^	Patient characteristics		
						Age	Gender	Attacks per 12 months
1	II	c.1396C > T,p.(Arg466Cys), rs28940870	1	Pathogenic	Not reported in ClinVar Mentioned in 12 manuscripts	63	Female	2
2	I	c.550G > A, p.(Gly184Arg), rs281875170	1	Pathogenic	Clinvar ID:79,144 Mentioned in 26 manuscripts	53	Female	48
3	I	c.1195C > T p.(Pro399Ser)	1	Pathogenic	Not reported in ClinVar (21,22)	55	Female	50
4	I	c.1312del, p.(Val438PhefsTer12)	1(2)	Likely pathogenic	Novel variant	34	Female	8
5						62	Female	10
6	I	c.1249 + 4A > G, p.?	1 (2)	Likely pathogenic	Novel variant	35	Female	60
7						58	Female	48
8	I	[GRCh38] chr11:g.57600729_57603011del	1	Pathogenic	Novel variant	32	Male	2

^a^If available ClinVar ID, and references if reported in few manuscripts, according to Mastermind.genomenon.com data [Access 18.06.2021]

## Discussion

Our nationwide study revealed an overall point prevalence of 0.53 per 100,000 inhabitants, which is lower than estimated in the literature (1 in 50,000 − 100,000 people worldwide) or other European studies (e.g., 1.54/100,000 in Sweden; 1.09/100,000 in Spain; 1.41/100,000 in Denmark; 1.54/100,000 in Italy; 1.55/100,000 in Austria) [1, 7, 11, 23–25]. Current data shows clear underdiagnosis of HAE in Latvia. Lack of awareness of HAE among physicians in Latvia, the intermittent nature of the symptoms, and non-specific signs of the disorder contribute to underdiagnosis [1]. The limited availability of C1-INH level and function assays in Latvia before 2021 may explain the low incidence of HAE, as well.

The percentage distribution of the data is only approximate due to the small study group.

In the study, we found that HAE was more common in females than males (90% vs. 10%). Accordingly, we confirmed that females have more frequent HAE attacks and more total sick days per year, which is similar to other reports [26, 27]. This might be explained by the fact that women are more likely to be symptomatic than men [27]. Perhaps women in Latvia also visit specialists more often, more persistently, and more patiently to clarify the diagnosis of HAE. Because only one male was affected, exact comparisons between genders cannot be made.

HAE type I was more prevalent than type II (70% vs. 30%, respectively), which is slightly different from the literature data (85% vs. 15%, respectively), but still comparable because type I dominates type II [3]. To exclude the diagnosis of acquired angioedema, C1q level and C1-INH autoantibodies were determined in two HAE type II patients without pathogenic variations in *SERPING1*.

In our study, a total of 10 patients were identified in eight unrelated families. In six families, diagnoses were confirmed molecularly in two cases with one or more symptomatic relatives available for genetic investigation. In four cases there were no data about other symptomatic relatives and healthy relatives were not available for the study to confirm de novo inheritance. HAE has an autosomal dominant pattern of inheritance, although it is estimated that 20% to 25% of cases are the result of spontaneous mutations in persons with no positive family history of the disease [28]. A family history of swelling is an important part of the diagnostic evaluation of HAE, but not an absolute requirement. In Latvia, no routine screening tests are performed on family members, including grandparents, parents, siblings, children, and grandchildren of HAE patients, as recommended in guidelines [5]. Therefore, it is possible that several HAE patients are not identified, especially asymptomatic patients or those with mild attacks of HAE during their lifetime. This is likely also due to limited availability of C1-INH level and function assays in Latvia before 2021. Family members of a patients with HAE should be screened more widely and more actively, especially when there is no longer limited availability for examinations of HAE in Latvia.

The median diagnostic time in our study was 20.5 years. Zanichelli et al. studied HAE patients from 11 European countries (Austria, Brazil, Czech Republic, Denmark, France, Germany, Greece, Israel, Italy, Spain, and the United Kingdom) and found an overall median diagnostic delay of 2.6 years, ranging from 0.13 to 17.3 years [29]. Diagnostic delay in Latvia is not acceptable and is longer than the rest of Europe. Diagnostic delay can be due to the rareness of HAE or to symptoms are being mistaken for other diseases, such as histaminergic angioedema and other causes of abdominal pain, such as acute appendicitis. The determination of diagnostic delay is important because patients who are symptomatic but have not yet been correctly diagnosed with the condition will be given inappropriate treatment or no treatment. Furthermore, delayed diagnosis leads to unnecessary investigations and procedures with economic, social, and psychological burden. Patients with a positive family history of HAE, if HAE had already been diagnosed in a family member, are less likely to experience diagnostic delay than patients without family history. Negative values in delay result from pre-symptomatic testing in some family members, as well as a shorter path to a correct diagnosis if symptoms have developed and are similar to those in a family member with HAE [23].

Forty percent of our patients reported more than 24 attacks in the previous year. There is no plasma-derived C1-INH in Latvia, which is currently the preferred long-term prophylaxis for the prevention of HAE attacks [5]. Only attenuated androgen (danazol and antifibrinolytic agent (tranexamic acid) are available in Latvia for long-term prophylaxis, which can be used as alternatives [5, 30].

During the previous year, 40% of patients had severe HAE attacks, similar to other studies [23, 31]. According to the international WAO/EAACI guideline, long-term prophylaxis should also be considered for these patients to reduce their attack frequency and severity [5].

During their lifetime, all patients have been hospitalised up to 20 times, resulting in social and economic hardship to the patient, patient’s family, health care system, and to society—just as elsewhere in the world [32, 33].

Subcutaneous oedema, lips, tongue, abdominal, and laryngeal oedema were the most frequent swelling locations, described in the other studies as the most common symptoms in HAE [23, 31].

Prodromes were reported by 90% of patients, which is slightly more than in other studies that reported prodromes between 68% and 82.5% [23, 34–36]. Our patients experienced prodromal symptoms such as tiredness, paraesthesia and/or pain, abdominal sensations, nausea, and *erythema marginatum*, also described in the literature as the most common prodromal symptoms in HAE [34, 36].

Factors that triggered attacks were mostly trauma, mental stress, surgical or dental manipulations, as were reported in other studies [31, 37, 38]. Before known triggers, short-term prophylaxis should be considered to avoid predictable HAE attacks [37].

[NO_PRINTED_FORM]Bradykinin B2 receptor-antagonist was effective treatment for HAE attacks and used by 90% of patients. Plasma-derived (pdC1-INH) was no longer available in Latvia at the time of the study. Fresh frozen plasma, tranexamic acid, opioids, and NSAIDs had poor effect during an HAE attack. Glucocorticoids and antihistamines (used by 80% and 100% of patients, respectively) before establishing the diagnosis had no effect during an HAE attack. These drugs are ineffective and are not beneficial for HAE [14]. Tranexamic acid and danazol, currently available for long-term prophylaxis in Latvia, had moderate effect. Fresh frozen plasma had poor effect for short-term prophylaxis. Even when a specific and effective treatment for HAE (recombinant C1-INH and Lanadelumab) has been registered and is available, governmental regulatory authorities and economic aspects of the health care system in Latvia have limited or delayed access. Unfortunately, the choice of maintenance treatment and prophylaxis drugs are currently limited in Latvia, reducing the opportunities for our patients to receive first-line therapy, as recommended in the guidelines [5]. Although effective medicines are available in the world, in Latvia alternatives are often used in the treatment of HAE, especially for long-term prevention, thus worsening the effectiveness of treatment in preventing HAE attacks. For this reason, there are still many patients with frequent and severe HAE attacks in Latvia. Use of drugs not specific for HAE is associated with and detrimental adverse effects [1].

Pathogenic variations of *SERPING1* gene were identified in 80% of all C1-INH-HAE patients. In 20% of patients with C1-INH-HAE, no pathogenic variations in *SERPING1* were found. Although pathogenic variations in *SERPING1* are the only known cause of the development of HAE types I and II, there are reported 5−10% symptomatic HAE patients with reduced C1-INH levels and/or functional activity in whom pathogenic variations cannot be found, even after carrying out a thorough genetic examination (including the entire *SERPING1* gene sequencing of the coding and non-coding parts of the gene and analysis of copy number variants) [39, 40]. In our study, we have even performed genome sequencing and genetic causes were still not identified in 20% of patients. From identified pathogenic variants, one was reported in ClinVar, two were reported in the literature, but three were reported for the first time in the HAE patients, according to available literature. Similarly, as in other reports, causative variants are mainly localised in the exons 7 and 8. Although we describe a small group of HAE type I and II patients, we did not observe differences in clinical symptoms between patients, similar to other authors who cannot give any clues about genotype/phenotype correlation [41, 42].

## Conclusion

This was the first ever nationwide survey of the point prevalence of HAE in Latvia, yielding 0.53 per 100,000 inhabitants. Current data shows clear underdiagnosis of HAE. We observed delay in diagnosis, even in those patients with a positive family history of angioedema. We suggest that lack of awareness of HAE among physicians, the intermittent nature of the symptoms, and non-specific signs of the disorder contribute to underdiagnosis, a delay in proper diagnosis, and thus undertreatment. Higher awareness and better information and communication between doctors would improve the diagnosis and management of HAE; as would screening of family members, patients with recurrent angioedema unresponsive to antihistamines and glucocorticoids, and patients with recurrent episodes of severe, unexplained abdominal pain. Timely HAE diagnosis and specific treatment can eliminate life-threatening attacks of this disease and significantly increase quality of life.

## Supplementary Information

**Additional file 1: Figure S1.** Family tree and timeline presentation of clinical characteristics, therapy, laboratory and genetic test results in a patient with hereditary angioedema. Patient 1. **Figure S2.** Family tree and timeline presentation of clinical characteristics, therapy, laboratory and genetic test results in a patient with hereditary angioedema. Patient 2. **Figure S3.** Family tree and timeline presentation of clinical characteristics, therapy, laboratory and genetic test results in a patient with hereditary angioedema. Patient 3. **Figure S4.** Family tree and timeline presentation of clinical characteristics, therapy, laboratory and genetic test results in a patient with hereditary angioedema. Patient 4. **Figure S5.** Family tree and timeline presentation of clinical characteristics, therapy, laboratory and genetic test results in a patient with hereditary angioedema. Patient 5. **Figure S6.** Family tree and timeline presentation of clinical characteristics, therapy, laboratory and genetic test results in a patient with hereditary angioedema. Patient 6. **Figure S7.** Family tree and timeline presentation of clinical characteristics, therapy, laboratory and genetic test results in a patient with hereditary angioedema. Patient 7. **Figure S8.** Family tree and timeline presentation of clinical characteristics, therapy, laboratory and genetic test results in a patient with hereditary angioedema. Patient 8. **Figure S9.** Family tree and timeline presentation of clinical characteristics, therapy, laboratory and genetic test results in a patient with hereditary angioedema. Patient 9. **Figure S10**. Family tree and timeline presentation of clinical characteristics, therapy, laboratory and genetic test results in a patient with hereditary angioedema. Patient 1.

## Data Availability

All data generated or analysed during this study are included in this published article and its Additional file information files.

## Abbreviations

HAE: Hereditary angioedema
C1-INH: C1-inhibitor
C1-INH-HAE: Hereditary angioedema with C1-INH deficiency
