# Supplementary material for: National survey on clinical and genetic characteristics of patients with hereditary angioedema in Latvia
Source: Allergy Asthma Clin Immunol. 2023 Apr 8;19:28. doi: 10.1186/s13223-023-00783-6 (PMC10082512; doi:10.1186/s13223-023-00783-6)
Supplement: Supplementary file 1 — Additional file 1: Figure S1. Family tree and timeline presentation of clinical characteristics, therapy, laboratory and genetic test results in a patient with hereditary angioedema. Patient 1. Figure S2. Family tree and timeline presentation of clinical characteristics, therapy, laboratory and genetic test results in a patient with hereditary angioedema. Patient 2. Figure S3. Family tree and timeline presentation of clinical characteristics, therapy, laboratory and genetic test results in a patient with hereditary angioedema. Patient 3. Figure S4. Family tree and timeline presentation of clinical characteristics, therapy, laboratory and genetic test results in a patient with hereditary angioedema. Patient 4. Figure S5. Family tree and timeline presentation of clinical characteristics, therapy, laboratory and genetic test results in a patient with hereditary angioedema. Patient 5. Figure S6. Family tree and timeline presentation of clinical characteristics, therapy, laboratory and genetic test results in a patient with hereditary angioedema. Patient 6. Figure S7. Family tree and timeline presentation of clinical characteristics, therapy, laboratory and genetic test results in a patient with hereditary angioedema. Patient 7. Figure S8. Family tree and timeline presentation of clinical characteristics, therapy, laboratory and genetic test results in a patient with hereditary angioedema. Patient 8. Figure S9. Family tree and timeline presentation of clinical characteristics, therapy, laboratory and genetic test results in a patient with hereditary angioedema. Patient 9. Figure S10. Family tree and timeline presentation of clinical characteristics, therapy, laboratory and genetic test results in a patient with hereditary angioedema. Patient 1. [file 13223_2023_783_MOESM1_ESM.pdf]

Additional file 1: figure S1. Family tree and timeline presentation of clinical characteristics, therapy, laboratory and genetic test results in a patient with hereditary angioedema. **Patient 1**

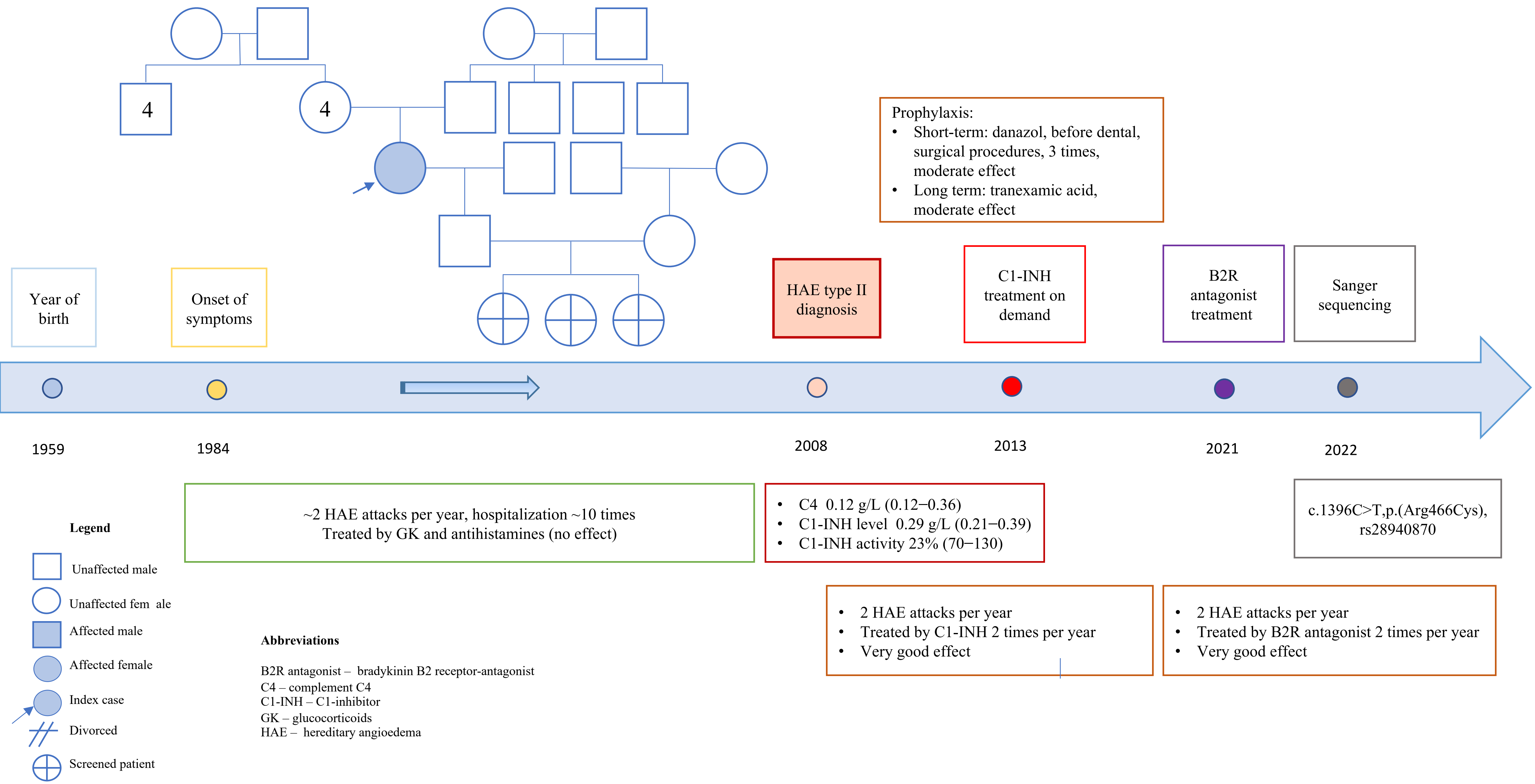

Additional file 1: figure S2. Family tree and timeline presentation of clinical characteristics therapy laboratory and genetic test results in a patient with hereditary angioedema **Patient 2**

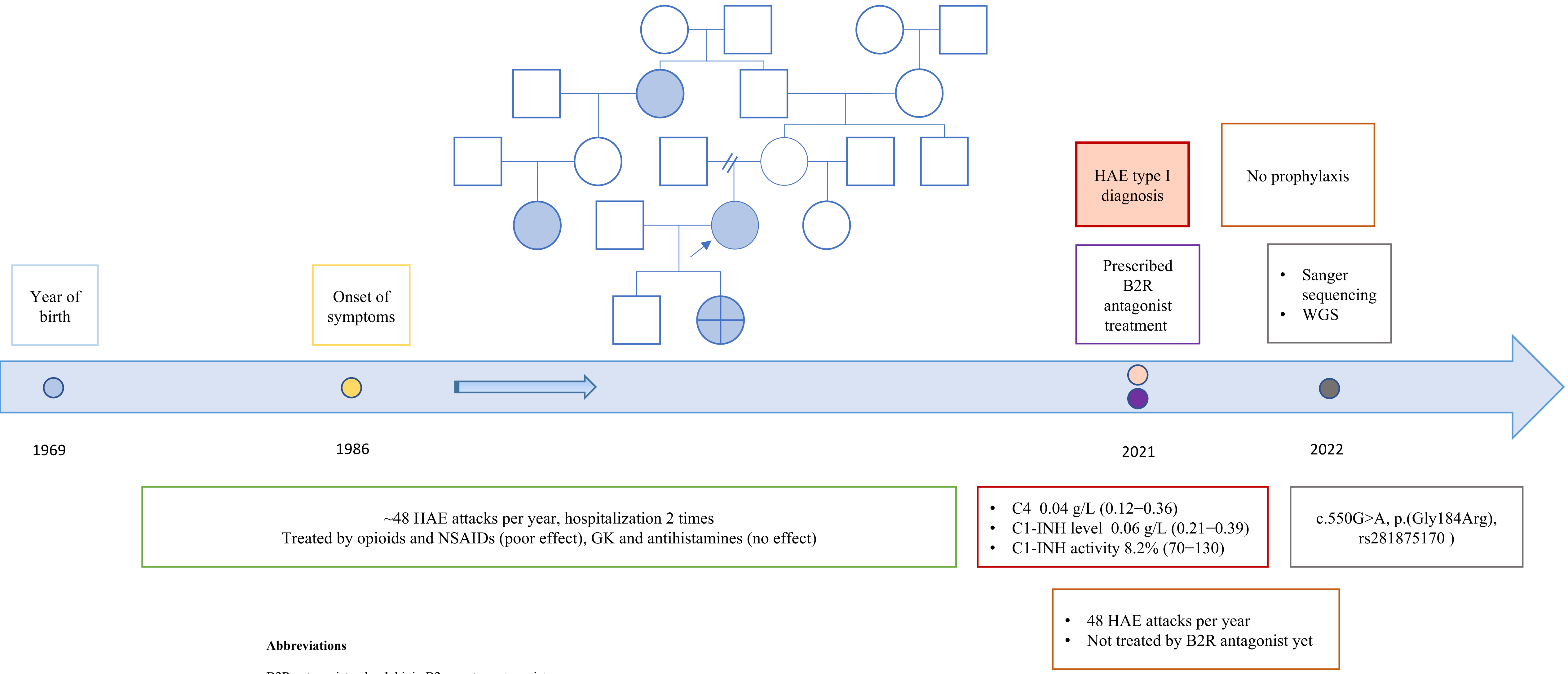

Abbreviations

B2R antagonist – bradykinin B2 receptor-antagonist
C1-INH – C1-inhibitor
C4 – complement C4
GK – glucocorticoids
HAE – hereditary angioedema
NSAIDs – non-steroidal anti-inflammatory drugs
WGS – whole genome sequencing

Additional file: figure S3. Family tree and timeline presentation of clinical characteristics, therapy, laboratory and genetic test results in a patient with hereditary angioedema. **Patient 3**

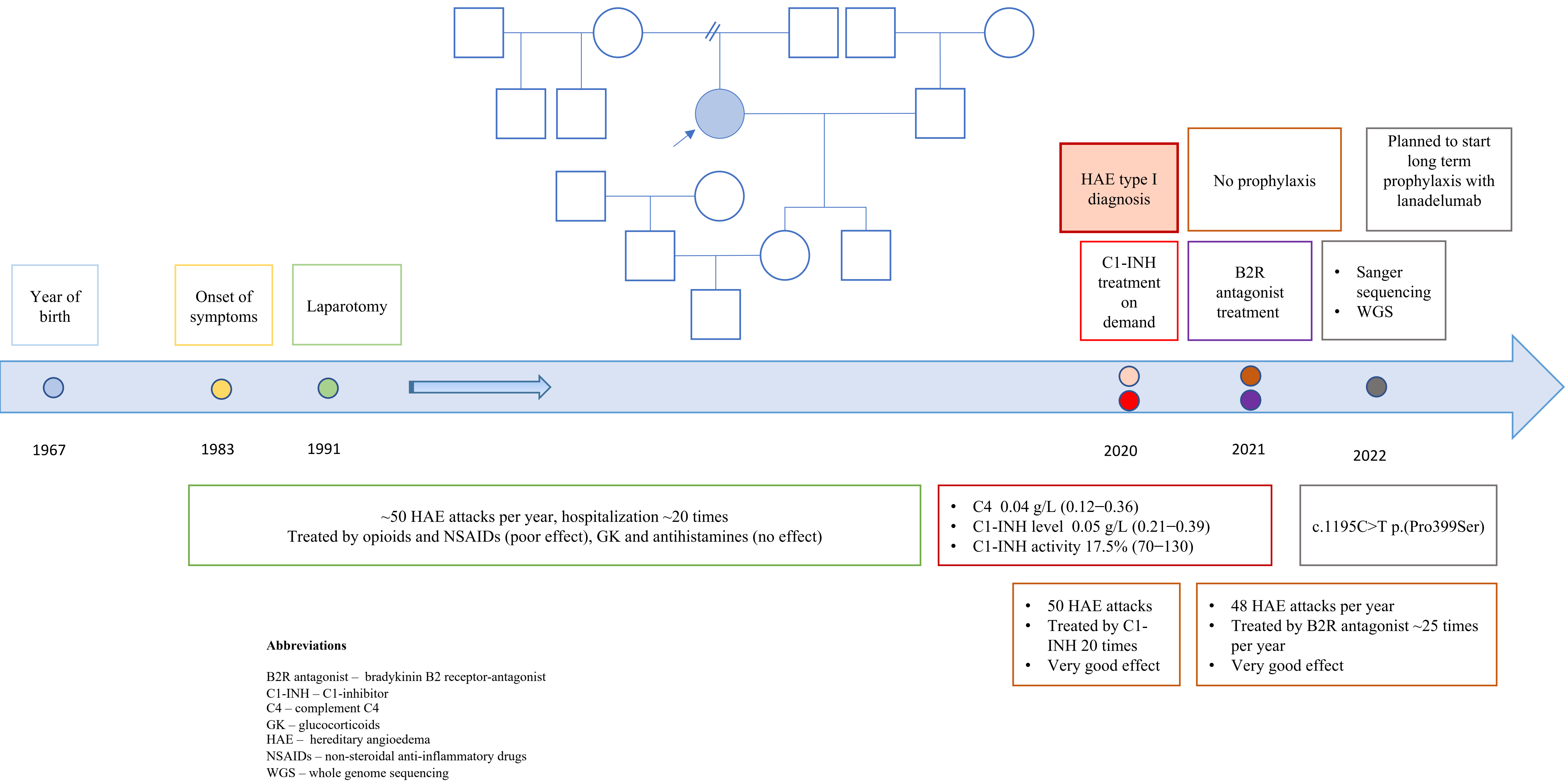

Additional file: figure S4. Family tree and timeline presentation of clinical characteristics, therapy, laboratory and genetic test results in a patient with hereditary angioedema. **Patient 4**

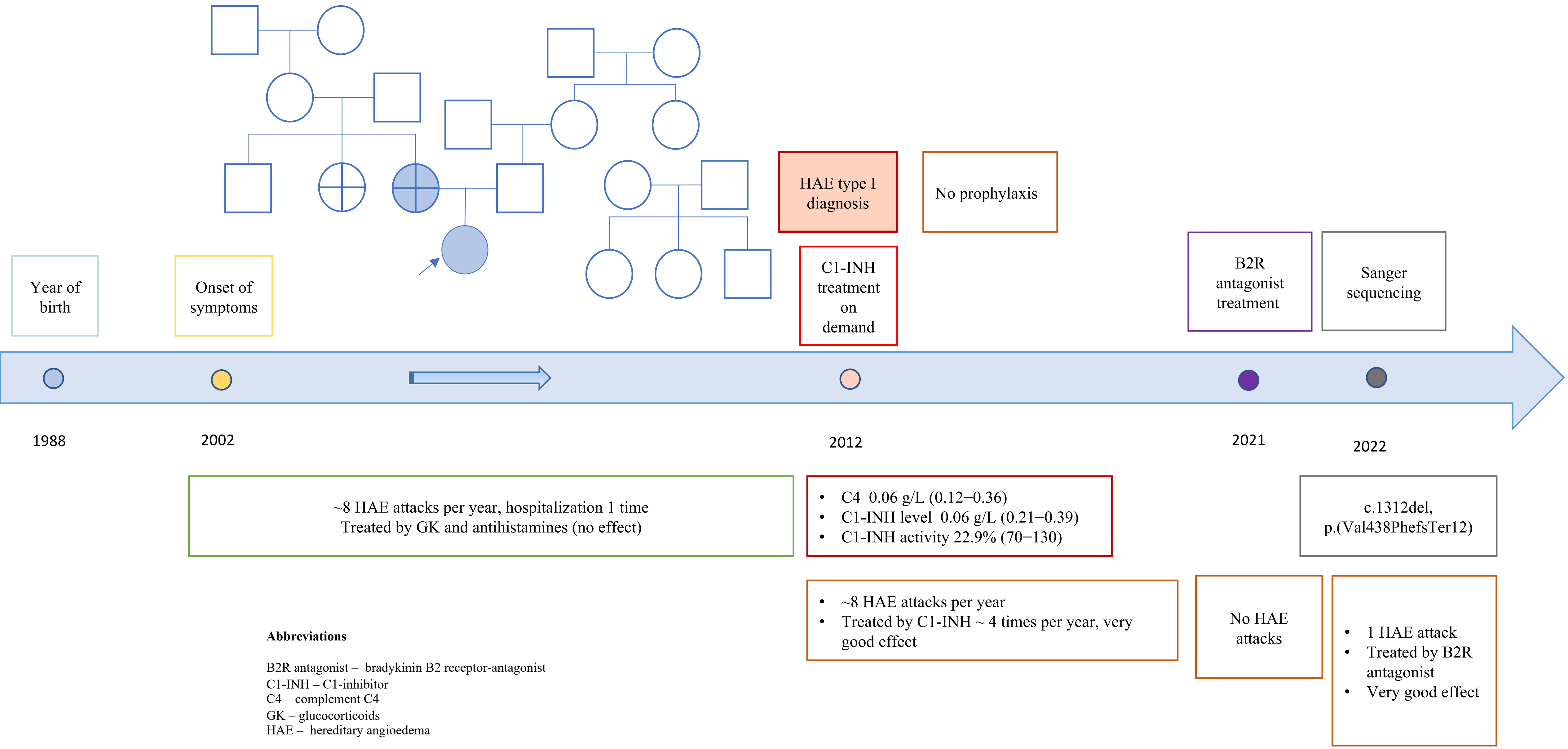

Additional file 1: figure S5. Family tree and timeline presentation of clinical characteristics, therapy, laboratory and genetic test results in a patient with hereditary angioedema. **Patient 5**

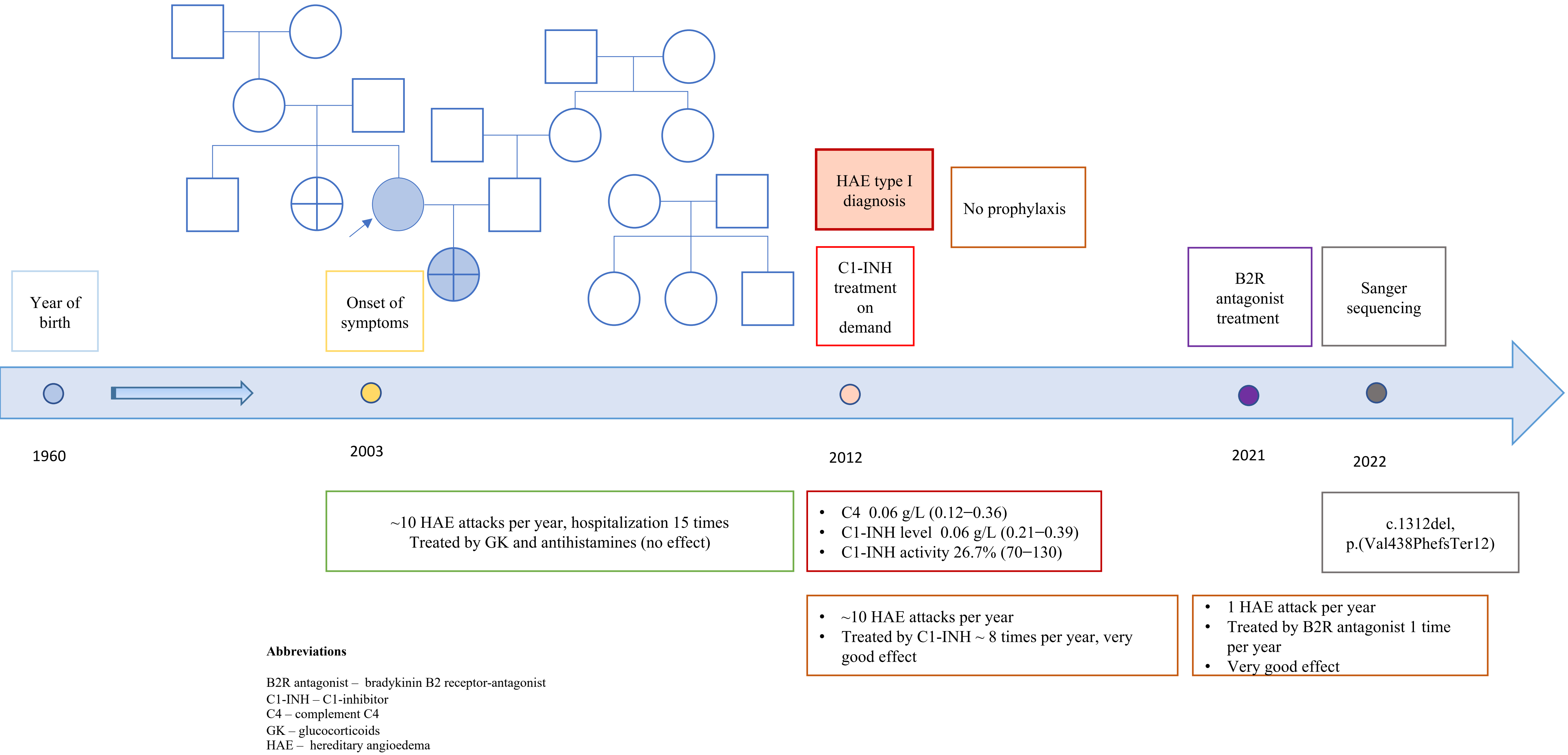

Additional file 1: figure S6. Family tree and timeline presentation of clinical characteristics, therapy, laboratory and genetic test results in a patient with hereditary angioedema. **Patient 6**

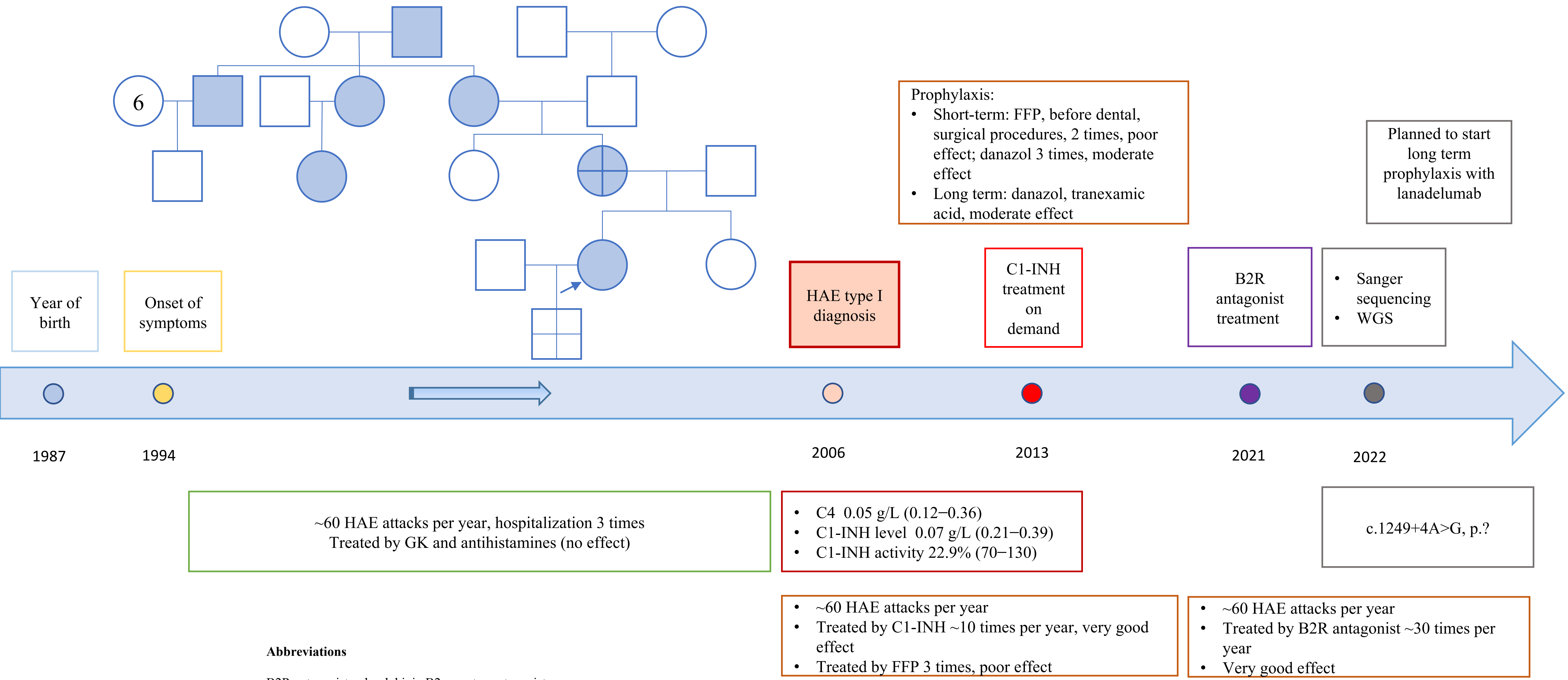

Abbreviations

B2R antagonist – bradykinin B2 receptor-antagonist
C1-INH – C1-inhibitor
C4 – complement C4
FFP – fresh frozen plasma
GK – glucocorticoids
HAE – hereditary angioedema
WGS – whole genome sequencing

Additional file 1: figure S7. Family tree and timeline presentation of clinical characteristics, therapy, laboratory and genetic test results in a patient with hereditary angioedema. **Patient 7**

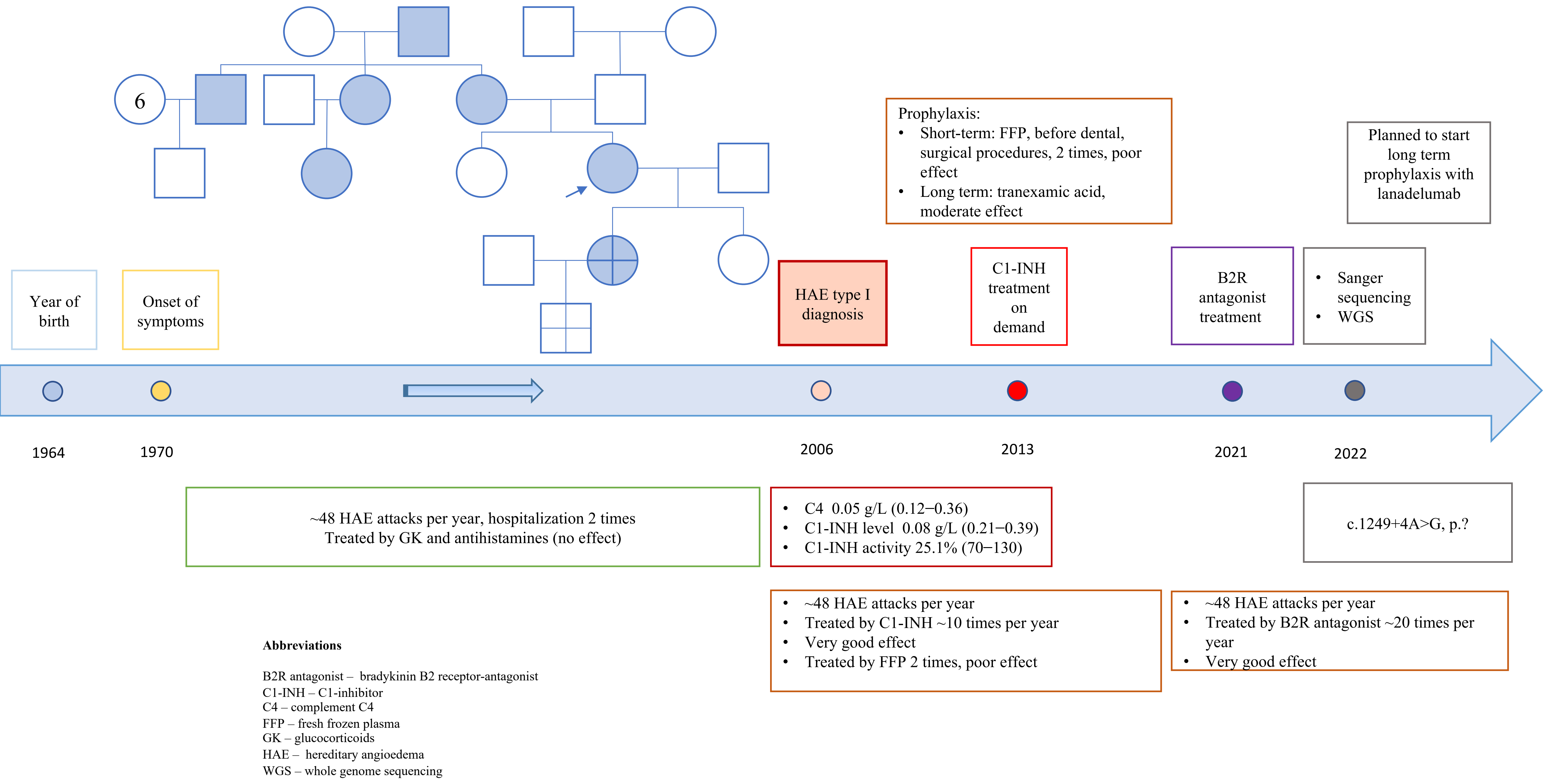

Additional file 1: figure S8. Family tree and timeline presentation of clinical characteristics, therapy, laboratory and genetic test results in a patient with hereditary angioedema. **Patient 8**

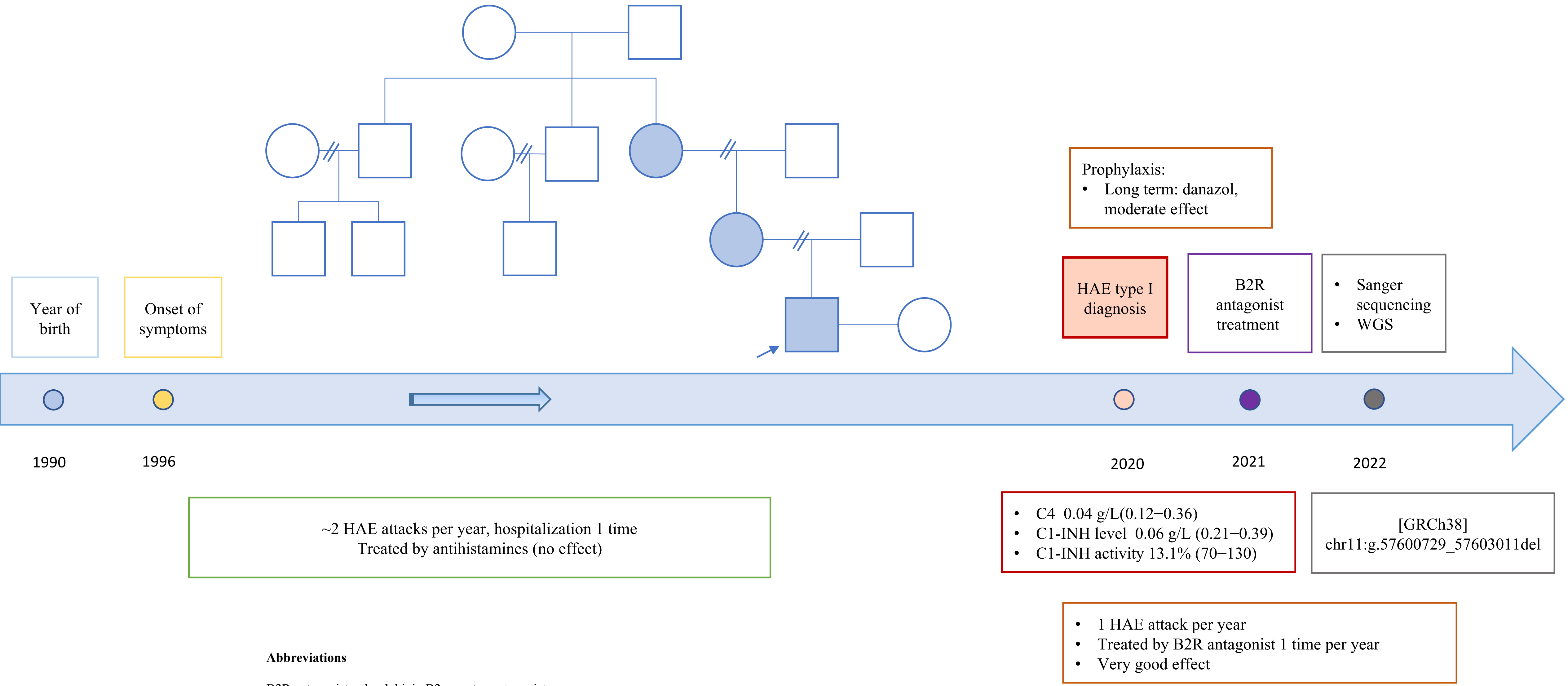

Abbreviations

B2R antagonist – bradykinin B2 receptor-antagonist
C1-INH – C1-inhibitor
C4 – complement C4
HAE – hereditary angioedema
WGS – whole genome sequencing

Additional file 1: figure S9. Family tree and timeline presentation of clinical characteristics, therapy, laboratory and genetic test results in a patient with hereditary angioedema. **Patient 9**

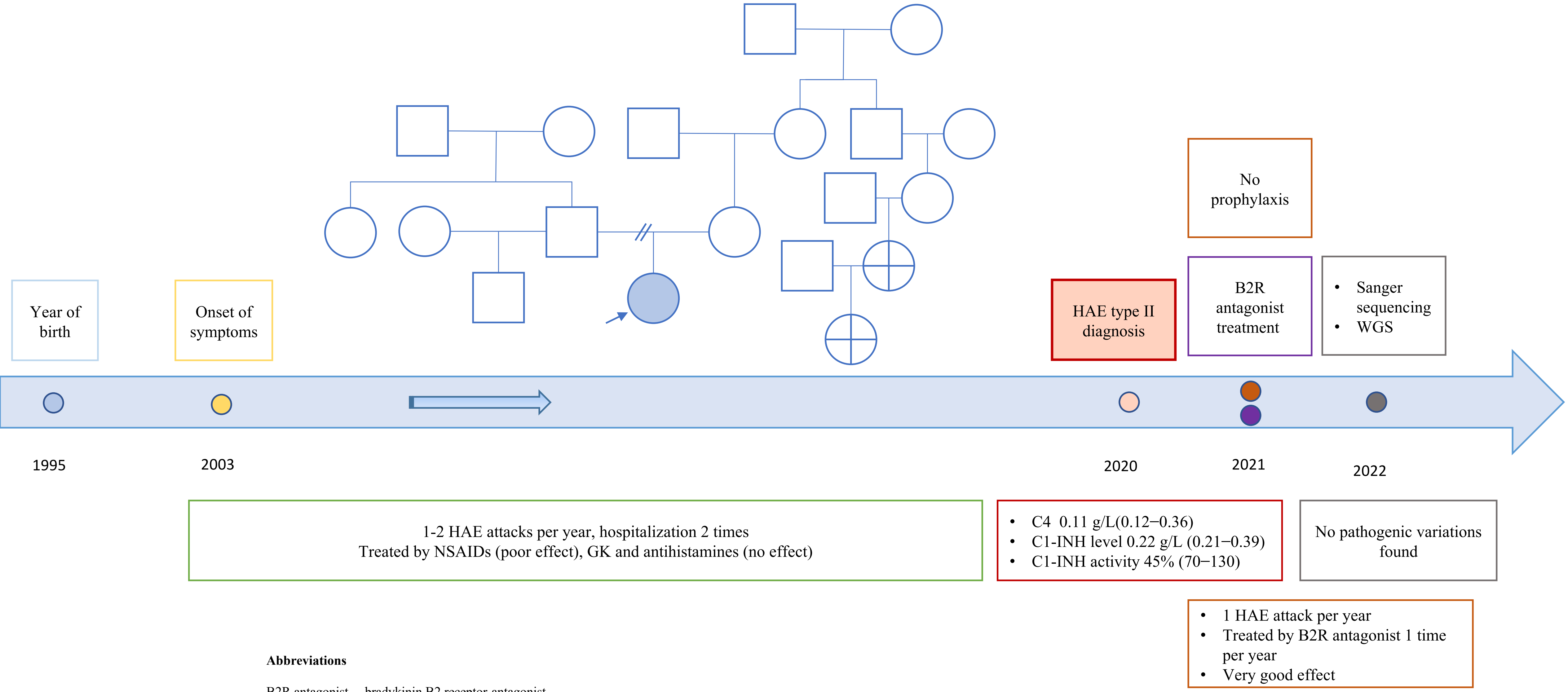

Abbreviations

B2R antagonist – bradykinin B2 receptor-antagonist
C1-INH – C1-inhibitor
C4 – complement C4
GK – glucocorticoids
HAE – hereditary angioedema
NSAIDs – non-steroidal anti-inflammatory drugs
WGS – whole genome sequencing

Addtional file 1: figure S10. Family tree and timeline presentation of clinical characteristics, therapy, laboratory and genetic test results in a patient with hereditary angioedema. **Patient 10**

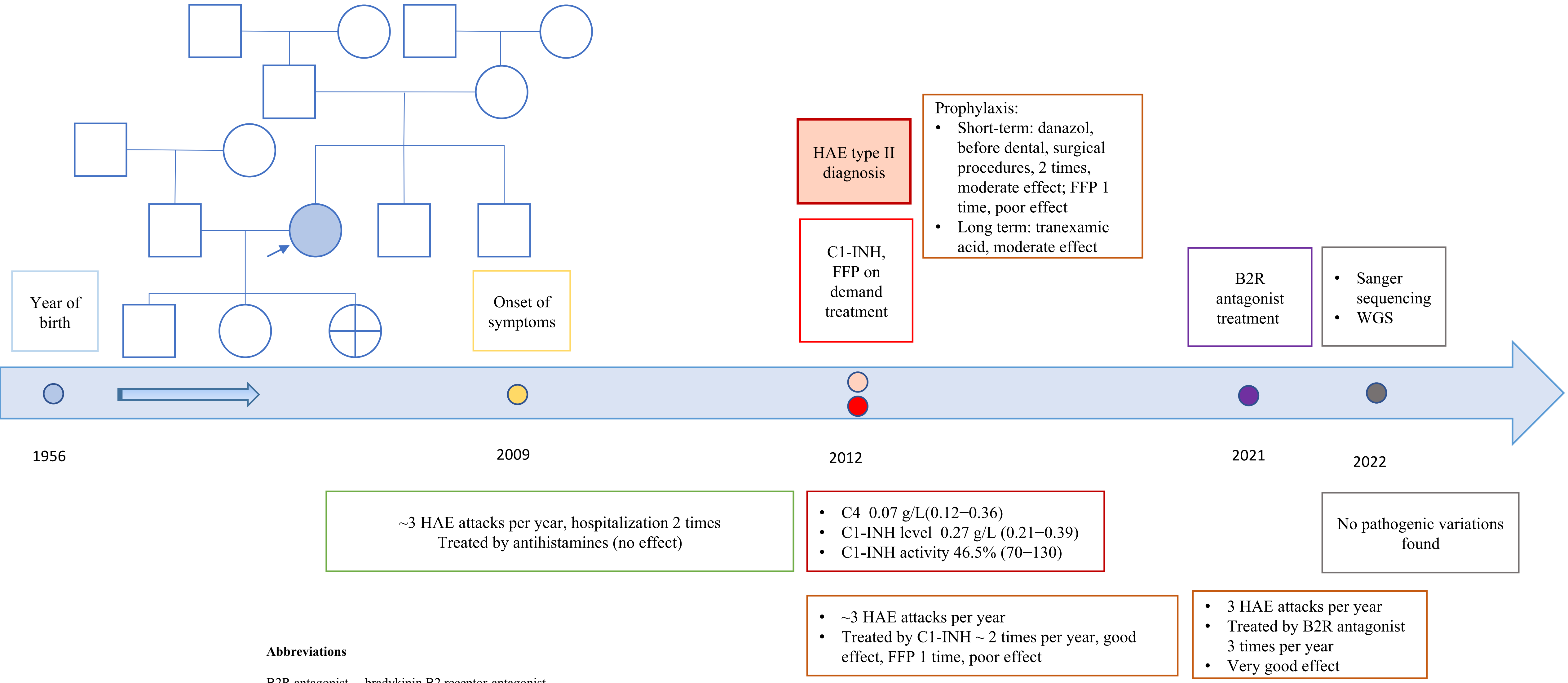

Abbreviations

B2R antagonist – bradykinin B2 receptor-antagonist
C1-INH – C1-inhibitor
C4 – complement C4
FFP – fresh frozen plasma
HAE – hereditary angioedema
NSAIDs – non-steroidal anti-inflammatory drugs
WGS – whole genome sequencing
